# Increasing Clinical Virulence in Two Decades of the Italian HIV Epidemic

**DOI:** 10.1371/journal.ppat.1000454

**Published:** 2009-05-29

**Authors:** Viktor Müller, Franco Maggiolo, Fredy Suter, Nicoletta Ladisa, Andrea De Luca, Andrea Antinori, Laura Sighinolfi, Eugenia Quiros-Roldan, Giampiero Carosi, Carlo Torti

**Affiliations:** 1 Institute of Biology, Eötvös Loránd University, Budapest, Hungary; 2 Ospedali Riuniti, Bergamo, Italy; 3 Policlinico di Bari, Bari, Italy; 4 Institute of Clinical Infectious Diseases, Catholic University, Rome, Italy; 5 National Institute of Infectious Diseases L. Spallanzani, Rome, Italy; 6 Ospedale S. Anna, Ferrara, Italy; 7 Institute of Infectious and Tropical Diseases, University of Brescia, Brescia, Italy; NIH/NIAID, United States of America

## Abstract

The recent origin and great evolutionary potential of HIV imply that the virulence of the virus might still be changing, which could greatly affect the future of the pandemic. However, previous studies of time trends of HIV virulence have yielded conflicting results. Here we used an established methodology to assess time trends in the severity (virulence) of untreated HIV infections in a large Italian cohort. We characterized clinical virulence by the decline slope of the CD4 count (n = 1423 patients) and the viral setpoint (n = 785 patients) in untreated patients with sufficient data points. We used linear regression models to detect correlations between the date of diagnosis (ranging 1984–2006) and the virulence markers, controlling for gender, exposure category, age, and CD4 count at entry. The decline slope of the CD4 count and the viral setpoint displayed highly significant correlation with the date of diagnosis pointing in the direction of increasing virulence. A detailed analysis of riskgroups revealed that the epidemics of intravenous drug users started with an apparently less virulent virus, but experienced the strongest trend towards steeper CD4 decline among the major exposure categories. While our study did not allow us to exclude the effect of potential time trends in host factors, our findings are consistent with the hypothesis of increasing HIV virulence. Importantly, the use of an established methodology allowed for a comparison with earlier results, which confirmed that genuine differences exist in the time trends of HIV virulence between different epidemics. We thus conclude that there is not a single global trend of HIV virulence, and results obtained in one epidemic cannot be extrapolated to others. Comparison of discordant patterns between riskgroups and epidemics hints at a converging trend, which might indicate that an optimal level of virulence might exist for the virus.

## Author Summary

The AIDS epidemic claims more lives per year than any other infectious disease, even though its cause, the Human Immunodeficiency Virus (HIV), is the youngest of all major human pathogens. The recent origin and great evolutionary potential of the virus raise the possibility that the virus might still be adapting to humans. Of primary interest is whether the virulence of the virus, i.e. its ability to cause disease, has been changing over time. Unfortunately, previous results have yielded conflicting results. We investigated time trends of virulence in the Italian HIV epidemic and found increasing virulence. The use of an established methodology allowed, for the first time, direct comparison with results obtained in other epidemics. The comparisons revealed that genuine differences exist in the trends of HIV virulence between different epidemics. Thus, there is no single time trend of HIV virulence worldwide. Our results are consistent with the hypothesis of increasing HIV virulence; however, clinical virulence combines viral and host factors, and the effect of host factors could not be excluded in our analysis.

## Introduction

Human immunodeficiency virus type 1 (HIV-1) is a recent pathogen of humans, estimated to have jumped to the human species about 80 years ago [1]. The virus may still be adapting to humans, and this evolution may affect also its ability to cause disease. There is evidence for heritable variation in viral traits that influence the virulence of the virus (severity of the infection) [2]–[5], which creates the conditions for the evolution of virulence. Multiple studies have attempted to assess time trends of HIV virulence, and have found increasing [6]–[11], stable [12]–[17], or decreasing [18],[19] virulence. The discrepancies may arise either from genuine differences between the study populations or from differences in the methodology used to quantify virulence. Comparing results from different studies and assessing global trends require the use of the same methodology in different geographical areas and epidemics. Here we use a methodology developed for an earlier analysis of the Swiss HIV Cohort Study [17], on an Italian cohort of comparable size. Our findings (increasing virulence in Italy vs. stable virulence in Switzerland) with the same methodology indicate that genuine differences exist between different epidemics. In addition, in the Italian cohort we found different patterns of virulence among the major exposure categories, indicating the possibility of relatively isolated sub-epidemics within the same country. Finally, we note that any measure of clinical virulence combines the effects of both host and virus factors, and must therefore be interpreted with caution. Our results are consistent with a time trend towards more virulent viruses (either by local evolution or by the introduction and spread of more virulent viruses from other epidemics); however, we could not exclude the effect of host factors.

## Results

### Time trend towards steeper CD4 slopes

The most direct measure of virulence available in untreated HIV infections is the rate of decline of the CD4 cell count in the blood. After a data selection procedure (see Methods), the CD4 slope could be calculated for 1423 patients. The earliest date of confirmed infection ranged between 1984–2005; the median number of CD4 counts used in the calculation of the slope was 9, the median baseline CD4 count was 596 cells/µL, and median age was 28.9 years at entry. The median CD4 slope was −50.4 cells/µL/year. The representation of the major exposure categories was as follows: intravenous drug-users (IDUs) 46.3%, heterosexuals (HETs) 36.6%, and men having sex with men (MSM) 17.1%; 31.9% of the patients were females. The CD4 slope displayed highly significant correlation with the date of confirmed infection (Spearman's rank correlation test: rho = −0.16, p<0.001; unadjusted effect: −1.7 cells/µL/year/year, 95% CI: −2.3–−1.1), in the direction of steeper CD4 slopes over the time span of the cohort. To account for potential confounding effects, we performed multivariate linear regression controlling for gender and exposure category (reference levels were male and IDU), age at the date of confirmed infection and baseline CD4+ cell count; the primary variable of interest was the date of confirmed infection. The distribution of the CD4 slopes had very long tails, with 10% of the data responsible for about 70% of the range. To approach normality, we therefore discarded outliers below the 5% and above the 95% quantiles. 1279 patients were included in the main analysis; the characteristics of this group are summarized in Table 1 according to exposure category. Best model fit was achieved after merging the HET and MSM exposure categories. The effect of the date of confirmed infection was highly significant (−2.0 cells/µL/year/year; 95% CI: −2.7–−1.3; p<0.001) in the regression analysis, confirming the time trend toward steeper CD4 slopes. The effect of date on the virulence markers in the main analyses is summarized in Table 2. The baseline CD4 count also had highly significant effect (−0.048 per year; 95% CI: −0.058–−0.039; p<0.001), indicating that the rate of loss slows down at lower CD4 counts, as has been observed before [7],[12],[17]. Furthermore, the HET and MSM exposure categories were associated with significantly faster CD4 decline compared with IDUs (−27.6 cells/µL/year; 95% CI: −50.4–−4.9; p = 0.017), and the interaction between the merged MSM/HET exposure category and the date of confirmed infection displayed borderline significance (0.90 cells/µL/year/year; 95% CI: −0.0–1.8; p = 0.056), indicating that the time trend may be less pronounced in these riskgroups. All other factors and interaction terms were dropped from the regression design due to lack of significance during model simplification (see Methods). To further investigate potential differences between the riskgroups, we carried out separate regression analyses restricted to each category. The effect of date was strongest in the analysis of IDUs (unadjusted effect: −1.7 cells/µL/year/year, 95% CI: −2.4–−1.1; adjusted effect: −2.0 cells/µL/year/year; 95% CI: −2.6–−1.4; p<0.001), weaker among HETs (unadjusted effect: −1.1 cells/µL/year/year, 95% CI: −2.0–−0.3; adjusted effect: −1.3 cells/µL/year/year; 95% CI: −2.2–−0.3; p = 0.008) and weakest in MSM (unadjusted effect: −0.6 cells/µL/year/year, 95% CI: −1.7–0.6; adjusted effect: −1.01 cells/µL/year/year; 95% CI: −2.16–0.14; p = 0.086), where it failed to reach significance. Factors for adjustment retained after model simplification were baseline CD4 count in all three riskgroups, and also gender and age in the HET exposure category (not shown).

**Table 1:** Demographic and clinical characteristics of the study groups included in the various analyses.

	**Exposure category**					
	**Heterosexual**		**IDU**		**MSM**	
**CD4 slope analyses**						
***Linear regression model***						
Number of patients	467		605		207	
Female	289 (61.9%)		128 (21.1%)		–	
	**Median**	**Interquartile range**	**Median**	**Interquartile range**	**Median**	**Interquartile range**
Date of confirmed infection	27/10/98	24/11/92–18/10/01	10/01/90	01/07/87–05/11/93	10/01/00	26/01/94–03/07/02
Age at confirmed infection (y)	30.80	26.08–38.36	26.17	23.27–30.18	31.41	26.75–39.65
Baseline CD4+ cell count (cells/μL)	600	472–742	585	449–796	567	480–735.5
CD4 slope (cell/μL/year)	−58.37	−96.42–−29.34	−42.4	−73.50–−19.89	−54.60	−93.90–−32.38
***Mixed-effect model***						
Number of patients	690		845		354	
Female	411 (59.6%)		174 (20.6%)		–	
	**Median**	**Interquartile range**	**Median**	**Interquartile range**	**Median**	**Interquartile range**
Date of confirmed infection	02/05/99	16/11/93–24/06/02	12/12/90	10/01/88–09/05/95	10/03/01	24/01/95–29/09/03
Age at confirmed infection (y)	31.86	26.53–39.68	26.60	23.57–31.05	33.02	27.19–40.81
Baseline CD4+ cell count (cells/μL)	563	428–717	562	414–775	560	459–728
**Viral setpoint analysis**						
Number of patients	400		157		228	
Female	202 (50.5%)		38 (24.2%)		–	
	**Median**	**Interquartile range**	**Median**	**Interquartile range**	**Median**	**Interquartile range**
Date of confirmed infection	15/10/01	10/01/00–17/09/03	28/11/00	27/10/98–13/01/03	26/10/02	07/02/01–07/05/04
Age at confirmed infection (y)	34.68	29.13–42.12	33.43	29.75–37.51	34.45	27.87–41.10
Baseline CD4+ cell count (cells/μL)	568.5	428–716.5	534	405–729	551.5	437–708
Setpoint (log10 RNA copies/mL)	4.17	3.73–4.55	4.00	3.26–4.40	4.35	3.93–4.66

The three virulence analyses could be performed on different, albeit overlapping, subsets of the cohort. In the early years, RNA measurements were not yet available, while recently enrolled patients do not have sufficient data points for the calculation of the CD4 slope. Statistics of the linear regression model of the CD4 slope refer to the subset stripped of outliers below the 5% and above the 95% percentiles; this subset was used in the regression analyses. The mixed-effect model accommodated more patients due to less stringent inclusion criteria. The small number of IDUs in the viral setpoint analysis reflects their diminished proportion in the cohort by the time RNA assays have become available.

IDU, intravenous drug users; MSM, men having sex with men.

**Table 2:** Estimated effects of the date of confirmed infection on the markers of disease progression.

Statistical model (dependent variable)	Number of patients	Estimate (per year)	95% CI	*p*
**Rate of CD4 decline** (cells/µL/year)				
Unadjusted effect of date	1423	−1.69	−2.29–−1.09	<0.001
***General linear model***	1279			
Effect of date		−2.00	−2.69–−1.31	<0.001
Date×(HET or MSM)		0.89	−0.02–1.81	0.056
***Mixed-effect model***	1889			
Effect of date		−1.86	−2.47–−1.25	<0.001
Date×MSM		1.10	−0.01–2.22	0.05
**Viral setpoint** (log10 RNA copies/mL)				
Unadjusted effect of date	785	0.062	0.038–0.085	<0.001
***General linear model***	785			
Effect of date		0.043	0.019–0.066	<0.001
**Rate of CD4 decline in patients with setpoint data** (cells/µL/year)				
Unadjusted effect of date	484	−3.71	−5.52–−1.90	<0.001
***General linear model***	484			
Effect of date (unadjusted for setpoint)		−4.42	−6.21–−2.63	<0.001
Effect of date (adjusted for setpoint)		−3.44	−5.19–−1.68	<0.001
***Mixed-effect model***	1120			
Effect of date (unadjusted for setpoint)		−3.85	−4.33–−3.37	<0.001
Effect of date (adjusted for setpoint)		−3.35	−3.85–−2.85	<0.001

General linear models of the rate of CD4 decline were fitted to the estimated CD4 slopes per patient; mixed-effect models were fitted to the original CD4 count data. Date refers to the earliest date of confirmed infection in each patient. Further co-factors included gender, riskgroup, baseline CD4 count, age and interaction terms; stepwise elimination of non-significant factors was performed based on likelihood ratio tests. Effects not related to date are not shown.

CI, confidence interval; HET, heterosexual; MSM, men having sex with men.

The analysis of the point estimates of the CD4 slope per patient cannot account for the misspecification of the slope of any one individual. To ensure reliable estimation, we required five CD4 measurements for the estimation of the slope; however, this requirement introduced a selection bias against fast progressors, who cannot accumulate sufficient data points before starting therapy. We therefore assessed time trends in the CD4 slope also with a mixed-effect model fitted to the original CD4 count data. The model included the same fixed effects as the general linear model, plus random intercept and slope to account for the correlation of repeated measurements within each individual. We included patients with at least three CD4 counts; this more relaxed selection criterion increased sample size to 1889 patients (the characteristics of this group are shown in Table 1). The results of the analysis were consistent with the findings from the linear regression model (Table 2).

### Time trend towards increasing viral setpoint

The magnitude of virus load in the blood after the resolution of primary infection (“viral setpoint”) has been shown to correlate with the rate of disease progression [20] and was therefore chosen as our second measure of virulence. Following data selection (see Methods), we calculated the setpoint for 785 patients as the mean log virus load in each patient. The date of confirmed infection ranged between 1996–2006; the median number of RNA measurements used for the calculation of the setpoint was 7, the median baseline CD4 count was 560 cells/µL, and the median age at entry was 34.1 years. The median setpoint was 4.21 log10 RNA copies/mL. The representation of the major exposure categories was as follows: heterosexuals 51%, MSM 29% and IDUs 20%; 30.6% of the patients were females. The characteristics of the study group are summarized in Table 1 according to exposure category. The setpoint displayed highly significant correlation with the date of confirmed infection (Spearman's rank correlation test: rho = 0.19, p<0.001; unadjusted effect: 0.062 log10 RNA copies/mL/year, 95% CI: 0.038–0.085), indicating an increasing trend over time. To account for potential confounding effects, we performed multivariate linear regression controlling for gender and exposure category (reference levels were male and HET), age at the date of confirmed infection and baseline CD4+ cell count; the primary variable of interest was the date of confirmed infection. The effect of the date of confirmed infection was highly significant (0.043 log10 RNA copies/mL/year, 95% CI: 0.019–0.066; p<0.001) in the regression analysis, confirming the time trend toward increasing setpoints. Higher baseline CD4 count was associated with lower virus load (−9.38×10^−4^ log10 RNA copies/mL/cell/µL, 95% CI: −1.25×10^−3^–−6.30×10^−4^; p<0.001) except among IDUs (IDU × baseline CD4 count: 0.001 log10 RNA copies/mL/cell/µL, 95% CI: 5.55×10^−4^–1.45×10^−3^; p<0.001). Female gender was associated with lower virus load (−0.24 log10 RNA copies/mL, 95% CI: −0.38–−0.11; p<0.001) and higher age correlated with lower virus load in the IDU exposure category (IDU × age at entry: −0.026 log10 RNA copies/mL/year, 95% CI: −0.036–−0.016; p<0.001). All other factors (including riskgroup) and interaction terms were dropped from the regression design due to lack of significance during model simplification. To further explore potential differences between riskgroups we carried out separate regression analyses restricted to each category. The effect of date proved strongest among MSM (unadjusted effect: 0.057 log10 RNA copies/mL/year, 95% CI: 0.019–0.096; adjusted effect: 0.050 log10 RNA copies/mL/year, 95% CI: 0.012–0.088, p = 0.001), weaker among HET (unadjusted effect: 0.047 log10 RNA copies/mL/year, 95% CI: 0.017–0.081; adjusted effect: 0.037 log10 RNA copies/mL/year, 95% CI: 0.004–0.065, p = 0.026) and weakest among IDU (unadjusted effect: 0.042 log10 RNA copies/mL/year, 95% CI: −0.023–0.108, p = 0.2; no factor had significant effect for adjustment), where it failed to reach significance. Factors for adjustment retained after model simplification included the baseline CD4 count for HETs and MSM, and gender in the HET exposure category (with female gender associated with lower setpoint as in the main analysis). Because the distribution of setpoints displayed considerable negative skew due to a long tail of low setpoints, we repeated all setpoint analyses after discarding low setpoint values to ensure normality. The results (including the effect of the date of confirmed infection) were consistent with those of the analyses performed on the complete dataset.

### Increasing setpoints contributed little to the time trend of CD4 slopes

We thus observed consistent trends of increasing virulence towards both faster CD4 decline and increasing setpoints during the time span of the cohort. Because higher setpoints have been associated with faster disease progression [20] and faster CD4 decline [17], we next asked to what extent increasing setpoints might have been responsible for the trend towards steeper CD4 slopes in our dataset. Estimates for both the CD4 slope and the viral setpoint were available for 484 patients. Consistent with earlier results, the CD4 slope was negatively correlated with the setpoint (Spearman rank correlation test: rho = −0.20, p<0.001; unadjusted effect: −11.8 cells/µL/year/log10 RNA copies/mL, 95% CI: −17.0–−6.6). To dissect the contribution of increasing setpoints and other time-dependent factors to the trend towards steeper CD4 decline, we performed an extended regression analysis of CD4 slopes by adding viral setpoint as additional explanatory variable to the earlier regression design. However, because this subset had a much later median date (due to the requirement of viral setpoint), we first tested the original multivariate regression design (without setpoint). The effect of the date of confirmed infection remained highly significant in this more recent subset (−4.4 cells/µL/year/year; 95% CI: −6.2–−2.6; p<0.001), and proved even stronger than in the main analysis. Further factors retained after model simplification included gender, baseline CD4 count, age and the interaction between gender and age. We then repeated the analysis including also setpoint as explanatory variable (the effect of age and the interaction between gender and age were dropped due to lack of significance in this design). The effect of setpoint proved highly significant (−14.4 cells/µL/year/log10 RNA copies/mL, 95% CI: −19.6–−9.3, p<0.001); however, the effect of the date of confirmed infection also remained significant (−3.4 cells/µL/year/year; 95% CI: −5.2–−1.7; p<0.001), and its strength decreased by only 22.2% compared with the analysis not controlling for setpoint. This result suggests that the bulk of the time trend towards steeper CD4 slopes was independent of variation (increase) in the setpoint. We tested also the inclusion of higher order terms in the setpoint into the regression model, but these proved not to be significant. Furthermore, calculating the expected effect of the increasing setpoints on the CD4 slopes by multiplying the unadjusted date coefficient of the setpoint (0.062 log10 RNA copies/mL/year, as obtained earlier) with the setpoint coefficient of the CD4 slope (−14.4 cells/µL/year/log10 RNA copies/mL) yields a result (0.89 cells/µL/year/year) that is consistent with the decrease in the effect of date (0.98 cells/µL/year/year) upon the inclusion of the setpoint in the analysis of the CD4 slopes. Using mixed-effect models, we could repeat the analyses on a considerably larger patient set due to the less stringent selection criteria (1120 individuals had three of more CD4 counts and an estimate for the setpoint), and the effect of date was consistent with the results of the linear regression analysis (Table 2). We thus conclude that the observed increase in the setpoints had a relatively small contribution to the time trend towards steeper CD4 slopes.

### Fine-scale patterns of virulence over time

**Figure 1:**
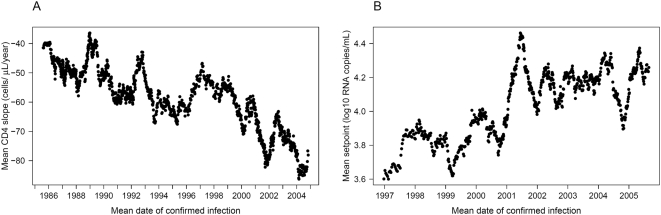
Moving averages of the CD4 slope and the viral setpoint. The figure reveals considerable short-term fluctuations over a steady trend towards steeper CD4 slopes (A) and higher setpoints (B). Each point represents the averaged value of 50 patients and is dated to their mean date of confirmed infection. The window of averaging moved along the list of patients sorted according to the date of confirmed infection: the first point represents the average of the first 50 patients, the second point represents the average calculated over the second through the 51^st^ patient, etc. The same datasets were used as in the multivariate regression analyses, i.e. outliers below the 5% and above the 95% quantiles were removed from the set of CD4 slopes.

**Figure 2:**
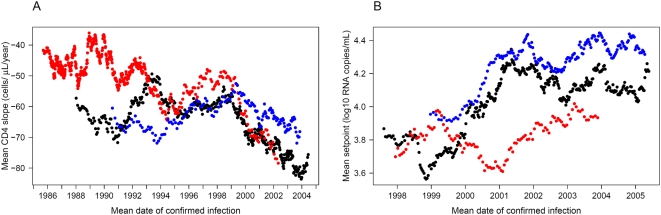
Moving averages of the CD4 slope and the viral setpoint per riskgroup. The figure shows time trends of the CD4 slope (A) and the viral setpoint (B) in the heterosexual (HET, black dots), intravenous drug-user (IDU, red dots), and men having sex with men (MSM, blue dots) exposure categories. This fine-scale representation suggests two stages in the time evolution of CD4 slopes within the riskgroups: in the first stage, IDUs lost their initial advantage of slower CD4 decline compared with HETs and MSM. After the convergence of the three categories, the second half of the observed epidemic has been characterized by a steady coupled trend towards steeper CD4 slopes in all groups. Trends in the viral setpoint seem to be strongly coupled between HETs and MSM with a recent deceleration of the increasing trend; the setpoint of IDUs seems to have fluctuated with no clear trend. Each point in the graphs represents the averaged value of 50 patients and is dated to their mean date of confirmed infection. The window of averaging moved along the list of patients sorted according to the date of confirmed infection: the first point represents the average of the first 50 patients, the second point represents the average calculated over the second through the 51^st^ patient, etc. The same datasets were used as in the multivariate regression analyses, i.e. outliers below the 5% and above the 95% quantiles were removed from the set of CD4 slopes.

Finally, to visualize short-term trends in the virulence markers, we plotted a moving average of both markers (Figure 1). Each point in the graphs represents the averaged value of 50 patients and is dated to their mean date of confirmed infection. The figure reveals that the trends towards increasing virulence (steeper CD4 slope and higher setpoint) have been stable throughout the time span of the cohort, with short-term fluctuations superimposed over the underlying long-term trend. We also calculated moving averages of both virulence markers in the major riskgroups separately (Figure 2). This fine-scale representation suggests two stages in the time evolution of CD4 slopes within the riskgroups: in the first stage, IDUs lost their initial advantage of slower CD4 decline compared with HETs and MSM. After the convergence of the three categories, the second half of the observed epidemic has been characterized by a steady coupled trend towards steeper CD4 slopes in all groups. This observation also explains why the difference in effect of date between riskgroups lost significance in the more recent set of patients for whom virus load data were also available. Trends in the viral setpoint seem to be strongly coupled between HETs and MSM with a recent deceleration of the increasing trend; the setpoint of IDUs seems to have fluctuated with no clear trend. Fine-scale resolution of time trends by moving averages thus allows a better interpretation of the results of the regression analyses. In particular, the slower CD4 decline of IDUs was restricted to the early years of the epidemic, and the estimated faster trend towards steeper slopes reflects the early convergence of this riskgroup to the others.

## Discussion

We found robust time trends both in the rate of CD4 decline and in the viral setpoint towards increasing virulence in untreated patients of the MASTER cohort. Over two decades of observations, the rate of CD4 decline in newly diagnosed infections has almost doubled, while the viral setpoint has been increasing at a rate of about one log per 16 years since RNA measurements had become available. The finding of increasing virulence is consistent with some [6]–[11], but not all [12]–[19] earlier studies of time trends of HIV virulence in various countries. Previously, it has been unclear whether discrepancies between studies of virulence arose from differences of how virulence was quantified, or they reflected genuine differences between geographical regions and cohorts. Importantly, we used a methodology adopted from an earlier analysis [17], which found stable levels of HIV virulence in Switzerland, over approximately the same time span as was analyzed in the current study. The use of the same methods ensures that the discrepancy reflects genuine differences in the temporal pattern of virulence between the Italian and the Swiss cohorts. This observation indicates that there is not a single “global trend” of HIV virulence, and results obtained in one local epidemic cannot be extrapolated to others. It also implies that correlating different markers of virulence will require the study of the same population with the different methods. Of particular interest would be the correlation of clinical markers, as used in this study, and replicative capacity [10],[19] as “top” and “bottom” measures of virulence. Replicative capacity is easy to measure for individual virus isolates; however, its relationship to pathogenic effects has been shown to be quite complex [21],[22].

We note that our “empirical” measures of virulence reflect the combined effect of both viral and non-viral (host or environmental) factors, and our findings are therefore not direct evidence for virus evolution. In addition to the factors incorporated in our analyses (gender, riskgroup, age), the progression of HIV infections has been shown to be influenced by host genetic factors [23], co-infections [24],[25] and socioeconomic factors [26]. Observed time trends could, in principle, reflect changes in any of these categories, and our data did not allow us to test or exclude the effect of such host factors. Our results are consistent with time trends in the viruses circulating in the cohort (either by evolution or by repeated introductions), but do not provide direct evidence or test for this hypothesis. The ultimate goal for future studies would be to identify viral factors contributing to virulence.

Transmitted drug resistance has been associated with faster CD4 decline in the first year of infection [27], the spread of resistance mutations might thus also have contributed to the observed trend in the CD4 slopes. However, the same trend existed in the pre-HAART era and most patients in our cohort were probably diagnosed after the first year of infection, which indicate the effect of other factors. We have also tested whether the criteria for starting therapy have changed over the time span of the cohort: the last CD4 count before the initiation of therapy showed a temporary peak around the beginning of the HAART era, but no trend over the studied period. A further potential confounding factor arises from our data selection procedure, which required three RNA measurements and five CD4 counts in untreated individuals to qualify for the estimation of the setpoint and the CD4 slope. These criteria selectively remove fast progressors, who may not have sufficiently long untreated disease history to accumulate the required number of measurements. However, such biased sampling should actually dampen a trend towards faster progressors and cannot have generated the observed trends as an artefact. Using mixed-effect models for the estimation of the CD4 slope, we were able to use less stringent selection conditions, and obtained consistent results. Finally, the earliest date of confirmed infection probably has a varying time lag compared to the true date of infection in the patients, which introduces variation in the disease stage at entry and uncertainty in the timing of infections. To minimize the effect of variation in the disease stage, we discarded the data points potentially associated with late-stage disease, and we also explicitly accounted for disease stage by controlling for the initial CD4 count. Uncertainties in the timing of infections may have contributed to short-term fluctuations: in particular, the temporary reversal of the CD4 slope trend around 1996 (Figure 1) might have resulted from an elevated proportion of slow progressors among the newly diagnosed cases due to increased willingness for testing after the introduction of HAART. However, the effect of fluctuations in the time lag must average out in the long run, and therefore cannot generate, nor hide long-term trends.

We also found differences in the time trends of virulence between riskgroups, which raises the possibility that the epidemics of the main exposure categories might have been founded by independent introductions of the virus. The strongest effect was the finding of a faster trend towards steeper CD4 slopes among IDUs, who initially had the slowest CD4 decline among the riskgroups. This suggests a plausible scenario that the IDU epidemic was by chance founded by a virus strain of reduced virulence, and occasional contact between the riskgroups later transmitted more virulent (and apparently fitter) viruses into this group. This hypothesis could be tested in the future by phylogenetic analysis. Alternatively, the increase in virulence might have reflected viral evolution within the IDU epidemic. It has been observed that explosive epidemics (characteristic of early spread in IDU populations) are associated with slow HIV evolution [28]. The IDU epidemic in our cohort followed the same pattern: more than half of all IDU infections were diagnosed in just six years between 1985–1990. Our findings are also consistent with a scenario that selection pressure to attain optimal virulence was weak in the early years of the rapidly expanding IDU epidemic, but intensified with the slowing of the expansion, driving virulence towards the levels already attained in the slower HET and MSM epidemics. Differences in the time trends of the viral setpoint were less pronounced between the exposure categories, and we have shown that the increasing trend of the setpoint had only a minor role in the time trend towards steeper CD4 decline.

Finally, we note that the overall median of the rate of CD4 decline was faster in the Swiss HIV Cohort Study (55 cells/µL/year) that had stable virulence than in the Italian MASTER cohort (50 cells/µL/year) that showed increasing virulence. This observation and the finding of converging levels of virulence among the riskgroups of the MASTER cohort are consistent with, but do not provide direct evidence for, the hypothesis that HIV has evolved close to the level of virulence that is optimal for its transmission [29], and local epidemics started by a founder virus of less-than-optimal virulence might experience an increase in virulence (whether by local evolution or repeated introductions). In contrast, epidemics founded by more virulent viruses might exhibit stable or even declining virulence.

We conclude that trends and patterns of HIV virulence may differ between geographical regions and even between different riskgroups within the same region. The results of individual studies can therefore not be extrapolated to predict the global patterns of the evolution of HIV virulence.

## Methods

### Study population and the calculation of virulence markers

The Italian MASTER (Management Standardizzato di Terapia Antiretrovirale) Cohort is a prospective longitudinal multicenter cohort comprising the general HIV patient population in referral centres throughout Italy. The analyses were restricted to patients of Italian nationality and white ethnicity for best representation of the local epidemics, and to the three major exposure categories: heterosexually infected patients (HET), intravenous drug users (IDU) and men having sex with men (MSM). Patients under age 15 were excluded from the analysis.

Markers of virulence were defined as in [17]. Briefly, we calculated two markers of virulence in antiretroviral naïve patients: the linear slope of the decline of the CD4 count and the mean log virus load (setpoint) of each patient. Serial measurements of CD4 cell counts and plasma RNA determinations for all registered patients were obtained from the MASTER database. Only data points preceding the first initiation of antiretroviral treatment in each patient were included in the analysis. The infections were dated to the earliest date of confirmed HIV-1 infection in the patients. To eliminate confounding effects of primary and late-stage infection, we discarded data points obtained within 200 days after the earliest date of confirmed infection in each patient, and data points obtained after the first CD4 count below 100 cells/µL. The viral setpoint was calculated in patients who had at least three remaining RNA measurements spanning at least 100 days, as the mean log10 virus load per mL. The CD4 slope was estimated by linear regression in patients who had at least five remaining measurements of the CD4 count, spanning at least one year. We have verified that fitting linear decline slopes yielded smaller residual sums of squared errors than fitting exponential decline. Analyses of the CD4 slope and the setpoint were restricted to patients who had their original first CD4 count or RNA measurement obtained within one year of the earliest date of confirmed infection.

### Statistical analysis

We estimated the effect of the date of confirmed infection on the virulence markers by analysis of covariance (general linear models). Categorical factors included gender and exposure category (riskgroup); reference levels were chosen to reflect the majority in the study groups. Continuous predictors included the earliest date of confirmed infection (converted to Julian date), age at the date of confirmed infection and the baseline CD4+ cell count, defined as the first CD4+ count obtained at least 200 days after the earliest date of confirmed infection. The starting models included the interaction between gender and riskgroup, and two-way interactions between all pairs of a continuous and a categorical predictor. Significant effects were assessed by stepwise elimination of non-significant interaction terms and non-significant factors, and finally by merging non-significant levels of categorical predictors [30].

The time trend of the CD4 slopes was also investigated with mixed-effect models fitted to the CD4+ T cell counts (relative to baseline) from all eligible patients over time; the models accounted for the correlation of repeated measurements within each individual by random intercept and time slope. The primary explanatory variable was time since the earliest date of confirmed infection (estimating the CD4 slope), and the models also included the interactions of the time with gender, riskgroup, the earliest date of confirmed infection, age at the date of confirmed infection, the baseline CD4+ cell count and (in some analyses) the viral setpoint, to account for the effect of these factors on the CD4 slope; and the 3-way interaction between time, date and riskgroup. Stepwise elimination of non-significant factors was performed based on likelihood ratio tests.

All statistical analyses were performed with the R software package (www.r-project.org).

### Moving averages

The datasets of both virulence markers were sorted into ascending order according to the date of confirmed infection of each patient. CD4 slope values below the 5% and above the 95% quantiles were excluded from the analysis. The average of 50 patients was calculated at a time, with the window of averaging moving along the lists of patients. The first point in each graph represents the average of the first 50 patients in the respective list; the second point represents the average of the patients second through 51^st^ in the list, etc. Each average was dated on the horizontal time axis to the mean date of confirmed infection among the respective 50 patients.
